# Distinguishing Protein-Coding from Non-Coding RNAs through Support Vector Machines

**DOI:** 10.1371/journal.pgen.0020029

**Published:** 2006-04-28

**Authors:** Jinfeng Liu, Julian Gough, Burkhard Rost

**Affiliations:** 1 Columbia University Bioinformatics Center, Department of Biochemistry and Molecular Biophysics, Columbia University, New York, New York, United States of America; 2 Columbia University Center for Computational Biology and Bioinformatics, New York, New York, United States of America; 3 Northeast Structural Genomics Consortium, Department of Biochemistry and Molecular Biophysics, Columbia University, New York, New York, United States of America; 4 Genome Exploration Research Group (Genome Network Project Core Group), RIKEN Genomic Sciences Center, RIKEN Yokohama Institute, Yokohama, Japan; The Jackson Laboratory, US; MRC-Harwell, UK; NHGRI-NIH, US; Lawrence Livermore National Laboratory, US; The Jackson Laboratory, US

## Abstract

RIKEN's FANTOM project has revealed many previously unknown coding sequences, as well as an unexpected degree of variation in transcripts resulting from alternative promoter usage and splicing. Ever more transcripts that do not code for proteins have been identified by transcriptome studies, in general. Increasing evidence points to the important cellular roles of such non-coding RNAs (ncRNAs). The distinction of protein-coding RNA transcripts from ncRNA transcripts is therefore an important problem in understanding the transcriptome and carrying out its annotation. Very few in silico methods have specifically addressed this problem. Here, we introduce CONC (for “coding or non-coding”), a novel method based on support vector machines that classifies transcripts according to features they would have if they were coding for proteins. These features include peptide length, amino acid composition, predicted secondary structure content, predicted percentage of exposed residues, compositional entropy, number of homologs from database searches, and alignment entropy. Nucleotide frequencies are also incorporated into the method. Confirmed coding cDNAs for eukaryotic proteins from the Swiss-Prot database constituted the set of true positives, ncRNAs from RNAdb and NONCODE the true negatives. Ten-fold cross-validation suggested that CONC distinguished coding RNAs from ncRNAs at about 97% specificity and 98% sensitivity. Applied to 102,801 mouse cDNAs from the FANTOM3 dataset, our method reliably identified over 14,000 ncRNAs and estimated the total number of ncRNAs to be about 28,000.

## Synopsis

There are two types of RNA: messenger RNAs (mRNAs), which are translated into proteins, and non-coding RNAs (ncRNAs), which function as RNA molecules. Besides textbook examples such as tRNAs and rRNAs, non-coding RNAs have been found to carry out very diverse functions, from mRNA splicing and RNA modification to translational regulation. It has been estimated that non-coding RNAs make up the vast majority of transcription output of higher eukaryotes. Discriminating mRNA from ncRNA has become an important biological and computational problem. The authors describe a computational method based on a machine learning algorithm known as a support vector machine (SVM) that classifies transcripts according to features they would have if they were coding for proteins. These features include peptide length, amino acid composition, secondary structure content, and protein alignment information. The method is applied to the dataset from the FANTOM3 large-scale mouse cDNA sequencing project; it identifies over 14,000 ncRNAs in mouse and estimates the total number of ncRNAs in the FANTOM3 data to be about 28,000.

## Introduction

The central dogma of molecular biology, first postulated by Francis Crick about 50 years ago [1], holds that genetic information is stored in DNA, that it is transferred into RNA through transcription by DNA polymerase, and that the information is finally decoded when RNA is translated into proteins. The paradigm has been that proteins largely constitute the machinery that makes life live: proteins carry out all structural, catalytic, and regulatory functions. In this view, RNAs mostly play the passive role of a “messenger.” When in vitro RNA splicing experiments revealed that RNAs can act as enzymes [2], this was initially considered an exception. Many more biological functions of RNAs are known today. RNAs can be divided into two classes: messenger RNAs (mRNAs), which are translated into proteins, and non-coding RNAs (ncRNAs), which are functional as RNA molecules rather than encoding proteins. The textbook examples of ncRNAs are tRNAs and rRNAs. ncRNAs have been found to carry out very diverse functions, from mRNA splicing (snRNAs) and RNA modification (snoRNAs) to translational regulation (microRNAs) and chromatin structure modulation *(XIST)* [3,4]. The functions of many ncRNAs remain unknown. During the first two phases of the Functional Annotation of Mouse cDNAs (FANTOM) project, only 17,594 of the 33,409 transcriptional units were determined to have coding potential; all remaining units were considered as putative ncRNAs [5,6]. The third phase of FANTOM [7] has discovered even more putative ncRNAs. Many of these ncRNAs are polyadenylated just like mRNAs; possibly more surprisingly, many of these ncRNAs are not short, rather they are, on average, even longer than protein-coding transcripts [6]. It has been estimated that 98% of the human genomic output may be ncRNAs, and speculated that differences in organism complexity may originate mainly from the vast difference in the amount of ncRNAs between higher eukaryotes and simpler organisms, rather than from the difference in protein-coding genes [8]. Given the importance of ncRNAs and the increasing body of large-scale data, the development of computational methods that distinguish between mRNAs and ncRNAs, i.e., between protein-coding and non-coding, becomes both increasingly urgent and increasingly feasible.

Few computational approaches have been specifically designed for the distinction between mRNAs and ncRNAs. Instead, methods developed for related tasks (detection of protein-coding regions within cDNAs, expressed sequence tags [ESTs], or prokaryotic genomic DNAs) have been applied to this task. The analysis of DNA alignments and codon usage is one of the most widely used strategies. CSTminer [9] identifies conserved sequence tags by comparing cross-species DNA alignments, based on the observation that in homologous regions, synonymous changes (i.e., base changes without amino acid substitution) occur more frequently than non-synonymous ones, and that non-synonymous substitutions often result in amino acid changes that preserve structural similarity. QRNA [10] adopts a similar strategy by using a hidden Markov model to identify ncRNAs. CRITICA [11] identifies protein-coding sequences in prokaryotic genomic DNAs through the combination of information from DNA alignments and from dicodon usage. Statistical models for mRNA constitute an alternative strategy. DIANA-EST [12] distinguishes coding ESTs from untranslated regions or out-of-frame cDNA windows with artificial neural networks, and ESTScan [13] detects coding sequences within ESTs through hidden Markov models. Alignments to known proteins in the databases were used by rsCDS [14] to identify coding regions in the FANTOM2 project [5]. Additional unpublished methods have also been used in the FANTOM3 distinction between protein-coding RNAs and ncRNAs (M. C. Frith, T. L. Bailey, T. Kasukawa, F. Mignone, S. K. Kummerfeld, et al., unpublished data). These include methods based on protein-domain-like regions from Pfam [15] and SUPERFAMILY [16,17], imposing a size threshold for longest open reading frame (ORF), and mTRANS, which uses both ORF length and cDNA coding potentials according to common features found in cDNA.

Most of the above methods succeed partially in the identification of protein-coding regions from cDNAs. However, the sustained performance in specifically distinguishing mRNA from ncRNA has not been thoroughly evaluated for any method because of the previous lack of data. Many methods were assessed using 5′ and 3′ untranslated regions as negative examples, which may not have the same characteristics as ncRNAs. Growing interest in ncRNA functions has prompted the construction of several databases cataloging different types of ncRNAs. Rfam [18] provides structure-annotated multiple sequence alignments for ncRNA families, most of which are structural ncRNAs. The major focus of Rfam is on the use of alignments and covariance models for automatically analyzing and annotating sequences, in analogy to Pfam [15]. However, for many Rfam sequences the direct experimental evidence for their transcription is missing. RNAdb [19] catalogs more than 800 experimentally studied mammalian ncRNAs, including microRNAs and snoRNAs, but not structural RNAs. NONCODE [20] contains more than 5,000 manually curated ncRNAs from 861 organisms, with more than 80% of the entries based on experimental data. It also classifies ncRNAs by their cellular function.

Here, we introduce CONC, a novel method for distinguishing between protein-coding RNAs and ncRNAs. The particular focus of CONC is on the reliable distinction of coding versus non-coding for long transcripts such as those abundantly identified by FANTOM3. Support vector machines (SVMs), like other supervised machine learning algorithms, try to learn decision rules from labeled input data (in this case, known protein-coding RNAs and ncRNAs) and use these rules to classify novel data. SVMs have a unique way of mapping input data into a very high dimensional feature space using kernel functions, and of identifying a hyperplane in this highly complex space that maximizes the distance from the closest samples to the hyperplane [21]. SVMs have been widely used for pattern recognition, as well as image and text classification. Recently, they have also been extensively applied to many problems in computational biology [22], including the detection of distant relatives of proteins [23], the prediction of subcellular localization [24,25], and the classification of microarray data [26]. In this study, we trained SVMs using eukaryotic ncRNAs from the RNAdb and NONCODE databases, and showed that protein features can be used to reliably distinguish protein-coding RNAs from ncRNAs. Sustained performance was demonstrated through rigorous cross-validation. When applied to the FANTOM3 data, the method estimated the number of ncRNAs in mouse to be about 28,000.

## Results/Discussion

### SVMs Using Proteins Features Were Excellent Classifiers for ncRNAs

Our major hypothesis for the development of our method was that native proteins have particular structural and sequence properties that distinguish them from putative translation products of ncRNAs. The protein characteristics that we selected included peptide length, amino acid composition, percentage of residues that were predicted by PROFsec [27–29] to adopt the secondary structure of alpha helix or beta strand, percentage of residues predicted by PROFacc [28–30] to be exposed to solvent, sequence complexity as measured by sequence compositional entropy [31], number of homologs from database searches, and sequence conservation computed as alignment entropy. We chose the longest possible translation products from each of the three forward frames, including those without obvious start codons. This approach was motivated by the attempt to account for sequencing errors: our method would be able to capture most of the protein regardless of the position of the error (immediately after the start codon or middle of ORF) and the type of the error (point mutation or insertions/deletion).

**Figure 1:**
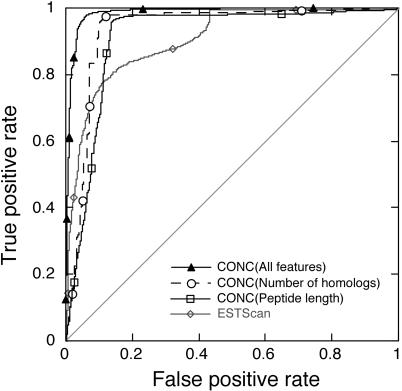
The ROC Curves for CONC A ROC curve plots the true positive rate (i.e., sensitivity) against the false positive rate (i.e., 1 − specificity). Shown are the ROC curves for CONC using all features, two of the top single features, and ESTScan. The diagonal line indicates random prediction.

One important measure of performance is provided by receiver operating characteristic (ROC) curves [32] that plot sensitivity against the value 1 − specificity over the full range of the specificity (Figure 1). The area under the ROC curve (the ROC score) describes the overall performance of the method under different thresholds. The ROC score of above 0.98 achieved by CONC (Figure 1) indicates an excellent performance. At the particular decision boundary chosen by a prediction method, the performance is more intuitively measured by specificity (Equation 1), sensitivity (Equation 2), or the harmonic mean of those two, known as the *F*-measure (Equation 3). Since the numbers of mRNAs and ncRNAs differ significantly in our dataset, reporting performance for both types (predicted mRNAs and predicted ncRNAs) becomes crucial (Table 1). At the threshold selected by our SVM classifier, protein-coding RNAs were predicted at *F* > 97% and ncRNAs at *F* > 94% (Table 1). We compared CONC with ESTScan [13], one of the publicly available methods that have been applied in the discrimination of ncRNAs. Run on our dataset with “–O –S” options so that only positive strands were analyzed, and using the default threshold of zero, ESTScan was about ten percentage points less accurate than our SVM-based method (Table 1). In contrast, using the same input features to train another machine learning algorithm, namely, a naïve Bayes classifier (see Materials and Methods), we were surprised to find that this simpler method yielded only slightly worse performance (Table 1).

**Table 1:**
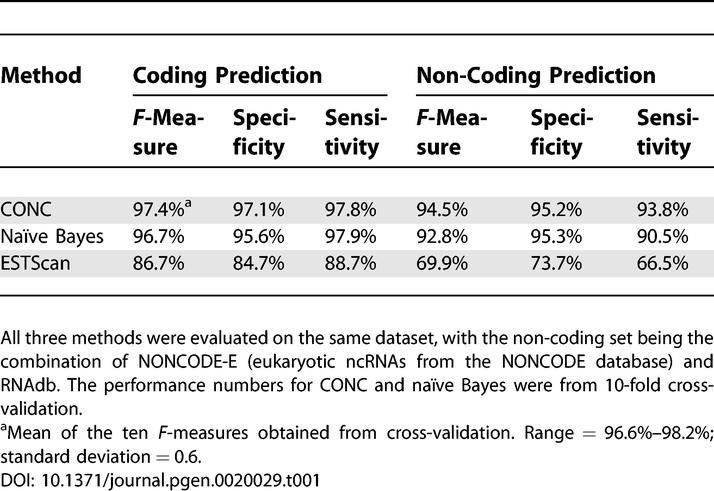
Prediction Performance of Different Methods

### All Features Helped, but the Combination Was Best and Most Robust

**Figure 2:**
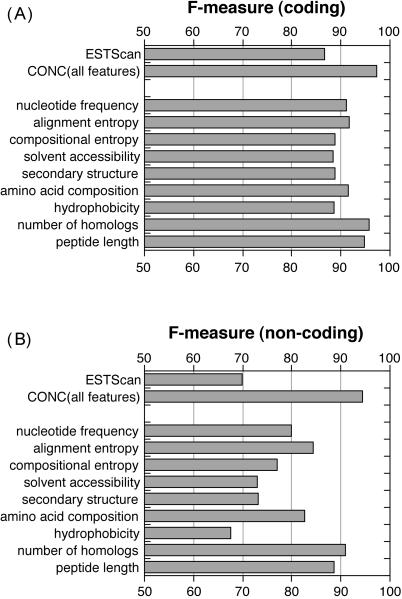
Performance of CONC with Different Input Features *F*-measures (harmonic mean of specificity and sensitivity; see Materials and Methods) were calculated for different SVMs for both the coding (A) and non-coding (B) predictions. Since the coding set was twice as big as the non-coding set, the percentage of incorrect predictions was bigger for the non-coding set, hence the smaller *F*-measures. When used individually, input features achieved *F*-measures of 67.6 to 90.9 on the non-coding set. Combining the features improved the performance to 97.4 for coding and 94.5 for non-coding. In comparison, ESTScan received *F*-measures of 86.7 and 69.9 for coding and non-coding predictions, respectively. The top-performing features were number of homologs in the protein database and peptide length.

We investigated the importance of individual input features by training separate SVMs on each feature. Most single features already discriminated coding from non-coding. However, the combination of all features performed much better than single features alone. The top-performing individual features were the number of database homologs and peptide length, followed by alignment entropy and amino acid composition (Figure 2). It is not surprising that the length or the number of homologs, alone, discriminate at reasonable accuracy. Most ncRNAs are short (the average length of ncRNAs was 526 nucleotides in our dataset, compared with 1,746 nucleotides for coding sequences), and having homologous proteins is certainly a very good indication for protein-coding. In fact, some methods exclusively rely on one of these two features: rsCDS [14] analyzed alignments with protein databases to make coding cDNA assignments for the FANTOM2 project, and “longest ORF” was also tested to evaluate ncRNAs in FANTOM3 (M. C. Frith, T. L. Bailey, T. Kasukawa, F. Mignone, S. K. Kummerfeld, et al., unpublished data). However, there are obvious limitations to these two features. Many ncRNAs (about 10% in our dataset) can be conceptually translated into peptides longer than 100 amino acids, and cDNAs coding for short proteins would also be incorrectly predicted as non-coding based on length alone. Similarly, protein-alignment-based predictions would err for cDNAs coding for novel proteins and ncRNAs that happen to align with some misannotated hypothetical proteins in the protein databases.

It was in light of these considerations that we chose to combine eight different protein properties, each of which has a good discrimination power by itself. The resulting combined SVM improved both specificity and sensitivity by more than three percentage points. The other advantage of combining all features is that predictions become more robust against mistakes in a single feature (e.g., incorrect alignment or incorrect peptide length). In such cases, other features can still contribute enough to the overall score and lead to correct predictions. For example, since our positive samples were taken from mRNAs that code for proteins in the Swiss-Prot database, it is trivial for them to find homologs in the databases. Consequently, SVMs using protein alignment alone are likely to perform worse on novel transcripts than on the well-defined datasets used to develop the SVM. In contrast, SVMs based on additional input features are likely to suffer less from this imbalance because they also rely on other signatures. Additionally, incorporating nucleotide frequencies (single nucleotide, dinucleotide, and triplet) into the multi-feature SVM slightly improved performance (about one percentage point) over the SVM with only protein-derived features.

### Most Predictions Were Very Reliable

**Figure 3:**
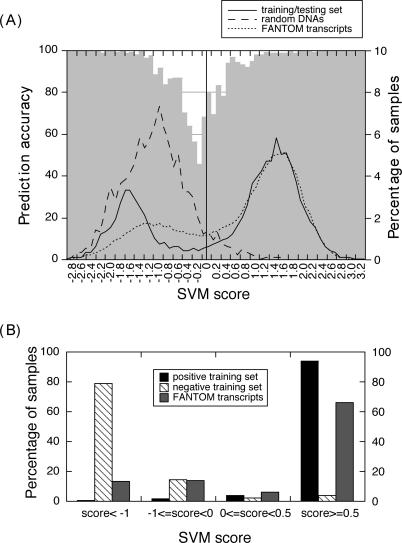
Distribution and Reliability of SVM Scores (A) The gray shading indicates prediction accuracy as a function of SVM score (left *y*-axis). Predictions are most accurate (>96%) when SVM scores are 0.5 or higher (for coding) or lower than −1 (for non-coding). Also shown are the score distributions (right *y*-axis) of three datasets: the training/testing set, random DNAs, and FANTOM3 transcripts. The solid vertical line in the middle indicates the SVM decision boundary (score = 0). (B) Most SVM predictions for the training/testing set are in the high specificity range. FANTOM3 transcripts have a slightly larger fraction of low accuracy predictions.

Prediction specificity and SVM output scores were highly correlated: the further the raw score was away from the SVM decision boundary (score = 0), the more accurate the prediction (Figure 3A). For the extreme ends of the score range, i.e., greater than 0.5 (coding) or less than −1 (non-coding), specificity exceeded 96%. As scores approached zero, specificity dropped sharply to about 50%, i.e., to levels reminiscent of random predictions. Therefore, the SVM output score is likely to be a good indicator for the reliability of novel predictions. Most predictions in our cross-validation tests fell into the region of high reliability (Figure 3A, solid line). Of all 5,601 positive samples in our dataset, 94% had output scores greater than 0.5, and 79% of the negative samples had a score less than −1 (Figure 3B). The score distribution was similar for random DNAs: the vast majority were reliably predicted as non-coding (Figure 3A).

We inspected a few cases for which CONC was very wrong indeed, i.e., ncRNAs with high scores and protein-coding sequences with very low scores. In many of the ncRNA cases, the experimental evidence for non-coding was ambiguous. For example, human transcript *ST7OT3* is part of a complex multi-transcript system at the *RAY1/ST7* locus. This locus contains two non-coding sense strand genes *(ST7OT3* and *ST7OT4)* that overlap with many alternative forms of the coding *RAY1/ST7* transcript, and two non-coding genes on the antisense strand *(ST7OT1* and *ST7OT2)* [33]. Although there was no explicit evidence that *ST7OT3* is protein-coding, the authors suggested it was possible. Another transcript from this locus was also annotated as non-coding in RNAdb (RNAdb ID LIT2007). However, according to GenBank's annotation, it seems that it is coding for ST7 isoform A, i.e., this may more likely be an example of a possible misannotation in RNAdb than of a serious mistake in our method. Another example was ncRNA *u1056* from NONCODE. This 7,291-nucleotide-long DNA encodes a partial coding sequence of the human DISC1 protein-coding gene and DISC2 ncRNA gene since it is the flanking region of the chromosome translocation breaking point associated with schizophrenia [34]. This is certainly not an example that the SVMs can learn! Most of the coding RNAs with very confident non-coding scores were very short proteins. The average protein length of the top ten mispredicted coding RNAs was 41 amino acids. Additionally, these short proteins had very few database homologs. They are likely to be the most difficult samples for any ncRNA classifiers. Some of the incorrectly predicted coding cDNAs encode proteins that are annotated as “hypothetical protein” in Swiss-Prot, i.e., they may not be coding after all. Overall, this detailed analysis appears to indicate that our estimates might be too conservative. However, the few cases for which we could analyze what appeared to be bad mistakes due to related experimental work did not suffice to make any general statement. Nevertheless, this detailed analysis did clearly underline the importance of the output score as a measure for prediction reliability.

The performance might not appear dramatically different between multi- and single-feature SVMs. However, the most important advantage of using multiple features became apparent when we tried to identify reliable output scores in single-feature-based predictions: we could not (Figure S1). In other words, there would have been no way to identify any subset of reliable ncRNA predictions in the FANTOM3 dataset had we only used single-feature SVMs.

### Mammalian ncRNAs Were More Difficult to Predict than Non-Mammalian

We trained SVMs on three different datasets of ncRNAs. The first dataset was 778 experimentally documented mammalian ncRNAs from RNAdb. The cross-validated SVM for this set identified 99% of the known protein-coding RNAs, while 14% of the ncRNAs were incorrectly predicted as coding (Table S1). When the SVM trained on this set was applied to the classification of eukaryotic ncRNAs from NONCODE and a set of randomly generated DNAs, the fractions of incorrect predictions were 8% and 9%, respectively (Table 2). We used random DNAs to assess the ability of the SVM to extract useful features that distinguish signals from random background noise. Our second training set comprised eukaryotic ncRNAs from NONCODE (set NONCODE-E). Cross-validation indicated a similar performance for predicting mRNAs, but a considerably lower false prediction rate for ncRNAs of around 5%. When this SVM was applied to the RNAdb set and random DNAs the error rates were 10% and 7%, respectively (Table 2).

**Table 2:**
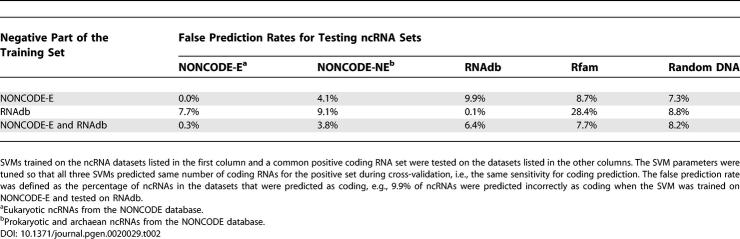
ncRNA False Prediction Rates for Different Datasets

The best SVM was trained on the ncRNAs from both RNAdb and NONCODE-E. This set included 1,158 mammalian ncRNAs and 1,512 non-mammalian eukaryotic ncRNAs. The specificity and sensitivity for the coding RNAs were similar to those for the other sets. However, the sensitivity for ncRNAs was notably higher when the SVM trained on this set was tested on RNAdb, NONCODE-NE (prokaryotic and archaean ncRNAs from NONCODE), and random DNAs (Table 2). A detailed analysis of the 166 incorrect predictions showed that 102 (61%) were from mammals and the remaining 64 from non-mammalian eukaryotes. The difference in performance indicated that the mammalian ncRNAs in RNAdb differed from those in NONCODE-E. On the other hand, SVMs trained on the combination of RNAdb and NONCODE-E appeared to capture the general features of ncRNAs, and performed well on all datasets, even on from NONCODE-NE. This was further confirmed by the high accuracy on Rfam (Table 2), which mostly contains rRNAs and tRNAs that are not included in RNAdb and NONCODE and which served therefore as a means of independent evaluation for the performance.

### ncRNA Predictions for FANTOM3

We applied CONC to 102,801 transcripts from the FANTOM3 project. Fourteen transcripts were shorter than 80 nucleotides. Since this was the threshold we used for our training, we automatically classified those 14 as non-coding. Of the remaining 102,787 transcripts, CONC predicted 28,316 to be non-coding. However, the fraction of highly reliable predictions was lower than that for our cross-validation set. The SVM output scores were in the reliable region of less than −1 for 13,873 transcripts (13.5%); another 14,443 (14.0%) were predicted as non-coding with lower confidence (Figure 3B). Our ncRNA predictions were in general agreement with FANTOM3′s non-coding annotation [7], most of which was done by using consensus from several previously developed computational methods. Of the 28,316 transcripts that we predicted as ncRNAs, 22,918 (80.9%) were annotated as non-coding. Among our confident predictions, 2,348 were not annotated as non-coding by the FANTOM consortium. This would be the most interesting set for experimental verification. The correlation between SVM scores and prediction accuracy provided an alternative means for estimating the number of non-coding cDNAs in the FANTOM3 dataset. More precisely, we could estimate the number of non-coding cDNAs based upon the correlation and the score distribution of the data. For example, if we knew that 1,000 samples scored between −0.7 and −0.6 and that 79% of the predictions were correct in this range, the number of ncRNAs and coding RNAs would be approximately 790 and 210, respectively. Based on this approach, we estimate the FANTOM3 dataset to contain 27,787 ncRNAs. By the same logic, we estimate that while most of these ncRNAs are in our set of ncRNA predictions, approximately 2,834 of them are likely in our set of protein-coding predictions.

Throughout the three FANTOM projects the importance of ncRNAs has become increasingly obvious, in particular for mammalian genomes. In parallel, detection methods have evolved. FANTOM3 topped the state of the art by combining many prediction methods through linear averaging (M. C. Frith, T. L. Bailey, T. Kasukawa, F. Mignone, S. K. Kummerfeld, et al., unpublished data). Here, we demonstrated that combining different protein features in SVMs improves performance significantly over single-feature SVMs. SVMs are an excellent framework for the efficient incorporation of different features; in fact, they are far more successful in accomplishing this than simple linear averages would be. Sometimes SVMs trained on different features are needed in different situations. For example, if the orientation of the input RNA is not clear, it may be better to use an SVM trained on protein features from all six frames (both forward and reverse). For FANTOM3 transcripts, orientation errors were rare; therefore, SVMs trained only on three forward frames identified 2,000 more ncRNAs than those trained on six frames. Presumably, most of these were antisense ncRNAs that were predicted as coding on the reverse frames. SVMs trained on protein alignment information are inevitably biased toward the known proteins. For predicting novel transcripts unrelated to known proteins it may therefore be advisable to avoid this bias by using SVMs trained without protein alignment features.

### Conclusions

We used a comprehensive collection of datasets for the evaluation of methods that distinguish between protein-coding RNAs and ncRNAs. Most of these data became available only recently. They were essential in the development of a novel prediction method that distinguishes between mRNA and ncRNA. Our method was essentially based on the assumption that features that capture general characteristics of native proteins will help considerably in this distinction task. We used SVMs to combine many relevant features that we chose initially largely by intuition rather than by optimization. The performance of our method appears to be significantly better than that of simpler methods. This suggests that our initial hypothesis about the value of protein-related features was largely correct. Performance may be further improved by incorporating other features into the SVM, such as output from ESTScan and other methods. The major strength of our multi-feature SVM is that it enabled the solution of two different tasks: (1) the annotation of the most reliable subset of ncRNAs in the FANTOM3 data (14,000), i.e., those that are most likely to be confirmed by more detailed, experimental follow-up studies, and (2) the estimation of the number of ncRNAs in the FANTOM3 set (28,000). The second task is very different from the first because our estimates for performance can be translated into estimated numbers but we cannot pinpoint exactly which 28,000 transcripts are indeed non-coding.

## Materials and Methods

### The dataset.

For protein-coding RNA, we first selected all eukaryotic proteins in the Swiss-Prot [35] database, and then removed sequence redundancy so that no protein pair in the set had a sequence similarity above an HSSP value of zero. The HSSP curve [36,37] relates alignment length to pairwise sequence identity or similarity; for alignments of 100 residues, HSSP = 0 corresponds to 33% pairwise sequence identity, and for alignments longer than 250 residues it corresponds to about 20%. cDNAs for these proteins were extracted from GenBank [38]. Potential sequence redundancy at the DNA level was further removed by running NCBI BLASTCLUST [39] with the “–L 0.7” option so that no sequence pairs were similar over 70% or more of their full length. Our final coding DNA set (positive set) contained 5,610 coding cDNAs.

Three eukaryotic ncRNA sets were used in this study as the negative set for training and testing: one from RNAdb [19], one from NONCODE [20] (named NONCODE-E), and the combination of the two. In all cases, sequences shorter than 80 nucleotides were excluded since potential translation products from these RNAs were too short for meaningful protein sequence alignments and secondary structure predictions. Sequence redundancy was removed in the same way as for the coding RNAs using BLASTCLUST. The final number of ncRNAs was 778 in the RNAdb set, 2,178 in the NONCODE-E set, and 2,670 in the combination set. We also tested our SVMs on three additional negative sets although they were not used during training. The Rfam set contained 29,009 nonredundant ncRNAs longer than 80 nucleotides from the Rfam database [18], the “random DNA” set included 2,000 randomly generated DNAs with length ranging from 80 to 3,000 nucleotides, and the NONCODE-NE (non-eukaryotes) set had 683 ncRNAs from prokaryotes and archaebacteria.

### Feature extraction and SVM training.

SVMs [21] are machine learning systems based on recent advances in statistical learning theory. In our study, we used SVM^light^ [40], a publicly available implementation of SVMs. The input features to the SVMs were the protein properties of potential peptides from RNAs. Specifically, each input RNA was translated into the three forward reading frames; the properties of the longest potential peptide from each frame were encoded into variables ranging from zero to one and were input to the SVMs. The following properties were used: (1) peptide length (four variables for length intervals of 20, 40, 80, and longer than 80 amino acids), (2) amino acid composition (20 variables), (3) average hydrophobicity [41] (one variable), (4) secondary structure content (percentage of residues in secondary structure classes helix, strand, and other as predicted by PROFsec [28–30]; three variables), (5) percentage of residues exposed to solvent as predicted by PROFacc [28–30] (one variable), (6) sequence compositional entropy [31] describing sequence complexity (one variable), (7) number of homologs from database searches using PSI-BLAST [42] against an in-house protein database (similar to NCBI's nr database) for four iterations with an *E*-value threshold of one (one variable), and (8) alignment entropy (the relative entropy between the observed fractions of amino acids and the respective background probabilities calculated for each position in the multiple alignment and averaged over the full length of the sequence; one variable).

In addition to these protein features, the frequencies of mono-, di-, and trinucleotides were also calculated for the entire input RNA and used as SVM inputs (84 variables). The total number of input variables was 180. These features were selected based on their distinguishing power when used alone in the SVMs. The SVMs were trained using the radial basis function kernel. The *g* parameter for the radial basis function kernel and the *C* parameter for the trade-off between training error and margin were determined by optimizing on a small subset of the training data. Cost factor *j* was set as the ratio between the number of negative training samples and the number of positive samples. The predictions from the SVMs were quite robust with regard to changes in kernel parameters (Figure S2). The largest feature weights determined by the SVMs are reported in Table S2.

### Evaluation of performance.

We tested the performance of our SVM classifiers via 10-fold cross-validation experiments. The dataset (coding plus non-coding) was randomly divided into ten equal-sized subsets. During each test, a SVM was trained on nine subsets and tested on the tenth one. This procedure was repeated ten times so that each subset was used as the test set exactly once. Performance was measured for each test, and the mean was reported. We also tested our method via 3-fold, 5-fold, 20-fold, and 50-fold cross-validation, and the results were virtually identical. The xi-alpha and leave-one-out estimates of the generalized error are reported in Figure S3.

Several measures were used to evaluate the performance of the SVMs. All of them were derived from the numbers of true positives (TP; coding RNA predicted as coding), false positives (FP; ncRNA predicted as coding), true negatives (TN; ncRNA predicted as non-coding), and false negatives (FN; coding RNA predicted as non-coding):

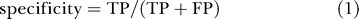


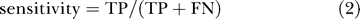


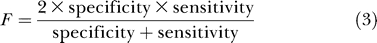



Specificity (also known to as accuracy or precision; Equation 1) and sensitivity (also known as coverage or recall; Equation 2) are the most commonly used measures. The harmonic mean of these two numbers (Equation 3), known as the *F*-measure [43], is often used as a single-value performance benchmark. Since both prediction categories (coding and non-coding) are of interest and the sizes of the two classes differ significantly, measuring performance for only one class is sometimes misleading and fails to capture the full picture; therefore, we reported the prediction performance for both classes in this study.

We also evaluated the SVMs using ROC curves [32]. SVM output scores were first sorted, and then the rate of true and false positives was calculated by setting the threshold to each score in the list. ROC curves plot the true positive rate as a function of the false positive rate. The area under the ROC curve (the ROC score) is the average sensitivity over all possible specificity values, which can be used as a metric for prediction performance over different thresholds. A totally random predictor will produce a curve around the diagonal line from bottom left to top right and will receive a score of about 0.5, while a perfect predictor will produce a curve along the left and top boundary of the square and will receive a score of one.

We compared our SVM with a naïve Bayes classifier downloaded from http://fuzzy.cs.uni-magdeburg.de/~borgelt/bayes.html. The naïve Bayes classifier was trained with default parameters using exactly the same input features and encoding as our SVM, and evaluated via the same 10-fold cross-validation procedure.

## Supporting Information

Figure S1 Prediction Reliability for Single-Feature and Multi-Feature SVMs For SVMs trained on all features (bottom right panel), there is a clear correlation between SVM output score and prediction accuracy: predictions are more accurate when the scores are further from the decision boundary (score = 0). For SVMs trained on single features (top panels), and to a lesser extent the one trained on two features (bottom left), there is little correlation, i.e., prediction accuracy can be very poor even when the SVM score is very far from zero.
(398 KB TIF)

Figure S2 SVM Parameter Optimization (A) Performance of the SVMs for different values of *g* in the radial basis function kernel when other parameters were fixed (*C* = 16; *j* = 0.5).
(B) Performance of the SVMs for different values of *C* (trade-off between training error and margin) when other parameters were fixed (*g* = 1; *j* = 0.5).
(215 KB TIF)

Figure S3 SVM Error Estimates Provided by SVM^light^
For each cross-validation run, the xi-alpha estimate (a pessimistically biased estimator) and the leave-one-out estimate of the generalized error were obtained from SVM^light^ output after the training. The leave-one-out estimate was similar to our reported error for 10-fold cross-validation.
(118 KB TIF)

Table S1 Cross-Validation Performance of SVMs Trained on Different ncRNA Sets (10 KB PDF)

Table S2 Feature Weights Determined by the SVM (12 KB PDF)

### Accession Numbers

The GenBank (http://www.ncbi.nlm.nih.gov/Genbank) accession number for human *ST7OT3* mRNA is AF400044, and the RNAdb (http://research.imb.uq.edu.au/rnadb) ID is LIT1900. The GenBank accession number for *u1056* is AF222983. The GenBank accession number for LIT2007 is NM_018412.

## Abbreviations

EST: expressed sequence tag
FANTOM: Functional Annotation of Mouse cDNAs
mRNA: messenger RNA
ncRNA: non-coding RNA
ORF: open reading frame
ROC: receiver operating characteristic
SVM: support vector machine
