# Supplementary figures and images for: Distinguishing Protein-Coding from Non-Coding RNAs through Support Vector Machines
Source: PLoS Genet. 2006 Apr 28;2(4):e29. doi: 10.1371/journal.pgen.0020029 (PMC1449884; doi:10.1371/journal.pgen.0020029)

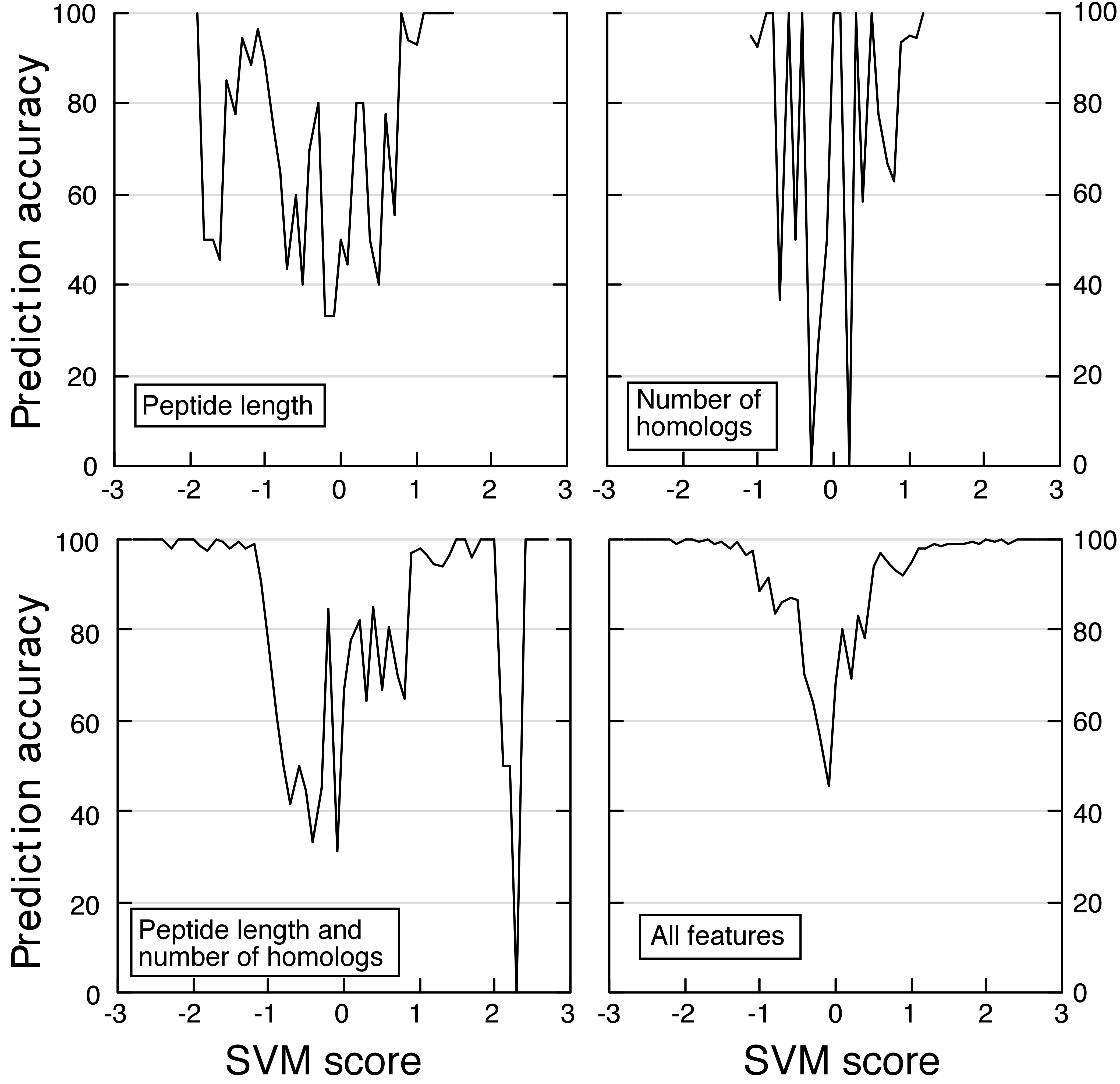

Supplement: Figure S1 — For SVMs trained on all features (bottom right panel), there is a clear correlation between SVM output score and prediction accuracy: predictions are more accurate when the scores are further from the decision boundary (score = 0). For SVMs trained on single features (top panels), and to a lesser extent the one trained on two features (bottom left), there is little correlation, i.e., prediction accuracy can be very poor even when the SVM score is very far from zero. (398 KB TIF) [file pgen.0020029.sg001.tif]

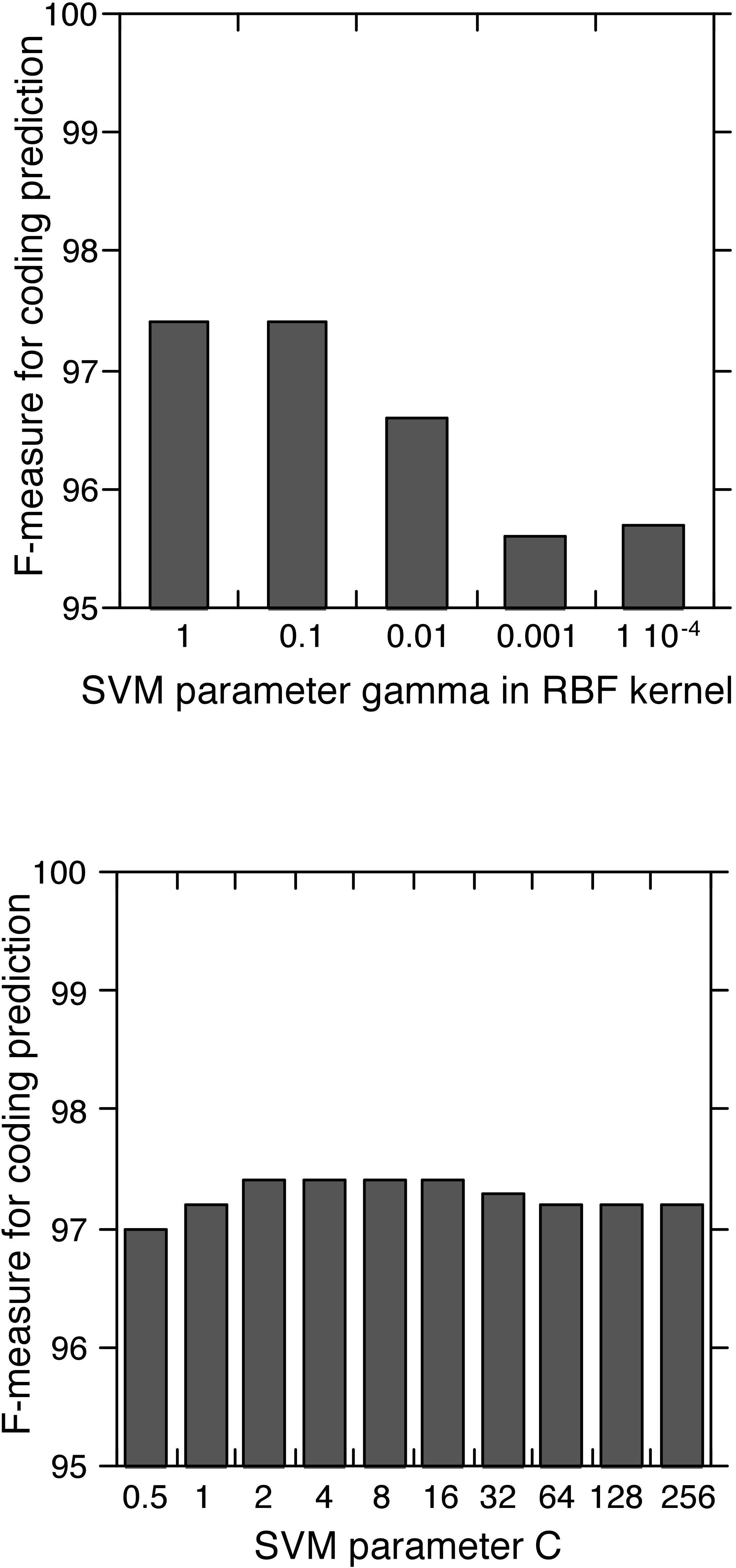

Supplement: Figure S2 — (A) Performance of the SVMs for different values of g in the radial basis function kernel when other parameters were fixed (C = 16; j = 0.5). (B) Performance of the SVMs for different values of C (trade-off between training error and margin) when other parameters were fixed (g = 1; j = 0.5). (215 KB TIF) [file pgen.0020029.sg002.tif]

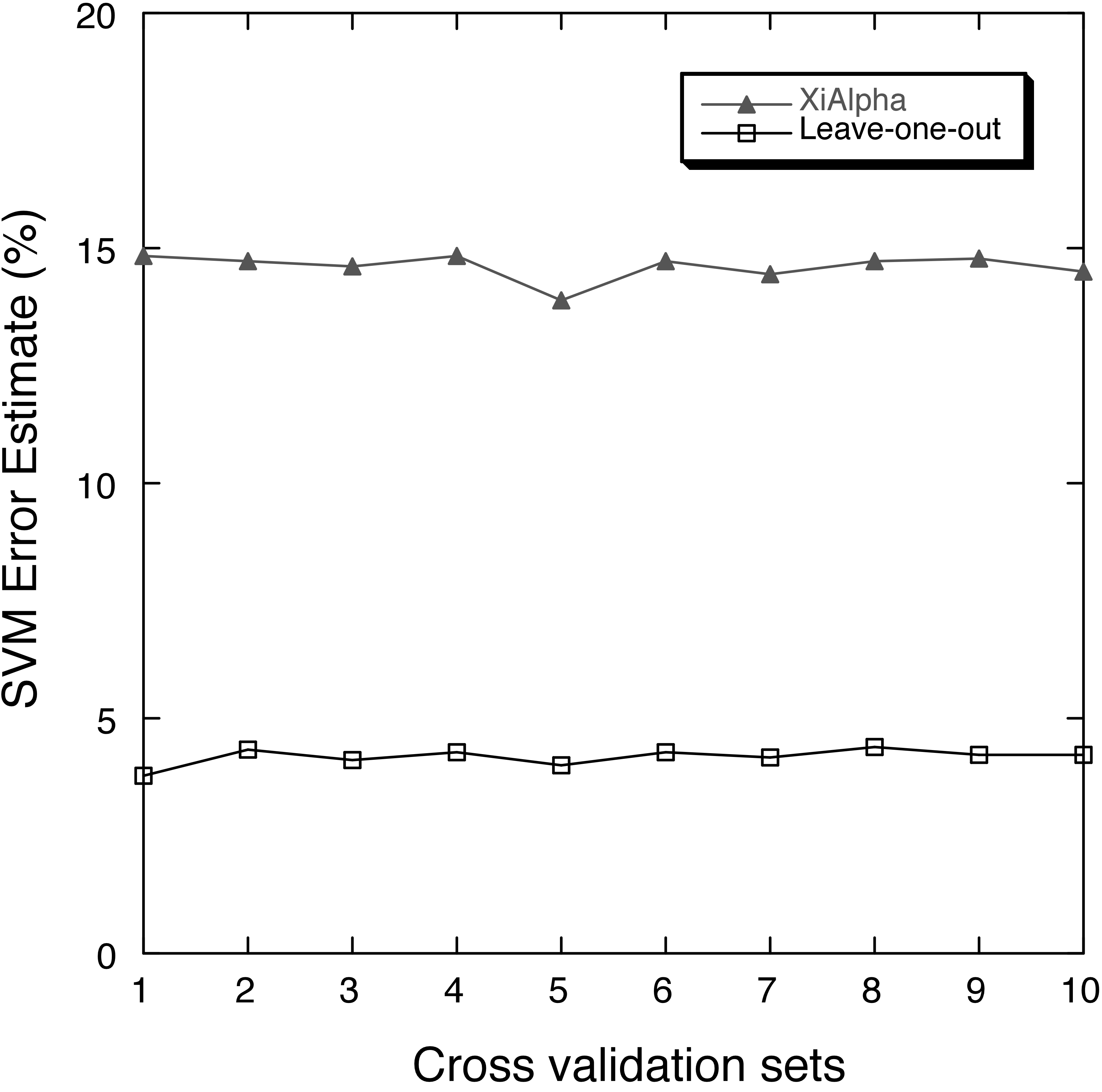

Supplement: Figure S3 — For each cross-validation run, the xi-alpha estimate (a pessimistically biased estimator) and the leave-one-out estimate of the generalized error were obtained from SVMlight output after the training. The leave-one-out estimate was similar to our reported error for 10-fold cross-validation. (118 KB TIF) [file pgen.0020029.sg003.tif]
