# Supplementary material for: Distinguishing Protein-Coding from Non-Coding RNAs through Support Vector Machines
Source: PLoS Genet. 2006 Apr 28;2(4):e29. doi: 10.1371/journal.pgen.0020029 (PMC1449884; doi:10.1371/journal.pgen.0020029)
Supplement: Table S1 — (10 KB PDF) [file pgen.0020029.st001.pdf]

Table S1: Cross-validation performance of SVMs trained on different ncRNA sets.

Negative part of the training set	Coding prediction			Non-coding predicition		
	F-measure	Specificity	Sensitivity	F-measure	Specificity	Sensitivity
NONCODE-E	98.2%	97.9%	98.4%	95.2%	95.8%	94.7%
RNAdb	98.4%	98.0%	98.7%	87.8%	90.2%	85.5%
NONCODE-E and RNAdb	97.4%	97.1%	97.8%	94.5%	95.2%	93.8%
