# Supplementary material for: Distinguishing Protein-Coding from Non-Coding RNAs through Support Vector Machines
Source: PLoS Genet. 2006 Apr 28;2(4):e29. doi: 10.1371/journal.pgen.0020029 (PMC1449884; doi:10.1371/journal.pgen.0020029)
Supplement: Table S2 — (12 KB PDF) [file pgen.0020029.st002.pdf]

Table S2: Feature weights determined by the SVM

Feature	Weight
Percentage of exposed residues in the longest peptide	-12.865
Frequency of di-nucleotide GG	-9.346
Frequency of nucleotide G	-8.090
Frequency of amino acid Gly in the second longest peptide	-5.197
Frequency of amino acid Trp in the longest peptide	-4.965
Number of homologues for the second longest peptide	16.508
Length of the third longest peptide	27.872
Number of homologues for the longest peptide	29.144
Length of the second longest peptide	38.411
Length of the longest peptide	45.452

Features with the five largest positive and negative weights are listed with their associated weights. The weights were calculated from the SVM^{light} model file as if it were from a linear kernel, using a script from the homepage of SVM^{light}. It should be noted that the weights are very sensitive to how the feature variables are normalized.
